# Burdening the poor: Extreme responses to COVID-19 in India and the Southeastern United States

**DOI:** 10.7189/jogh.10.020327

**Published:** 2020-12

**Authors:** Kanika Sharma, Kathryn M Yount

**Affiliations:** 1 Department of Sociology, Emory University, Atlanta, Georgia, USA; 2 Hubert Department of Global Health and Department of Sociology, Emory University, Atlanta, Georgia, USA

India and the United States – both large, diverse, and unequal democracies – have responded very differently to the COVID-19 pandemic. Yet, in both cases, the poor have been disproportionately burdened. In India, a hurried and unplanned lockdown caused extreme distress among workers and poorer sections of society while social protections have been woefully inadequate. In the US, working classes and racial minorities are bearing the brunt of COVID-19 infections and deaths. Precipitous actions to reopen the economy, and overt references to workers as ‘human capital stock’ [1], are exposing a vulgar and partisan mindset, in which productivity ‘trumps’ both equity and humanity.

In India, more than 2 850 000 COVID-19 cases and more than 54 000 deaths are confirmed [2]. Yet, age-sex-disaggregated data are lacking, and there is little hope of accessing data by caste, religion, or occupation. Compounding the direct health impacts of COVID-19, India’s national lockdown with just four hours of notice has had its greatest impact on the most vulnerable [3]. Trains had stopped even before the lockdown was declared. Hundreds of thousands of migrant workers were stranded, without transportation to return home. As work and savings dried up, they began long, arduous journeys back home, often walking long distances with children and older dependents in tow. Mass unemployment, food insecurity, inadequate health care, and stalled transportation created nothing short of a humanitarian crisis.

In the absence of timely and reliable vital statistics, the authors have documented lockdown-related deaths through media reports [4]. To date, at least 906 deaths are directly attributable to the national lockdown. Because only deaths reported in the media are counted, the actual number of deaths caused by India’s lockdown is likely to be higher. The causes of these deaths range from starvation and financial distress, to exhaustion, vehicular or train accidents during migration, lack or denial of medical care, suicides, police brutality, crime, and alcohol-withdrawal-related deaths and suicides. A majority of documented deaths were of daily wage labourers and migrant workers. To take just one example, a 12-year-old female agricultural laborer, belonging to an indigenous community, walked over 100 km for three days [5]. She died of exhaustion 11 km from her village in central India.

**Figure:**
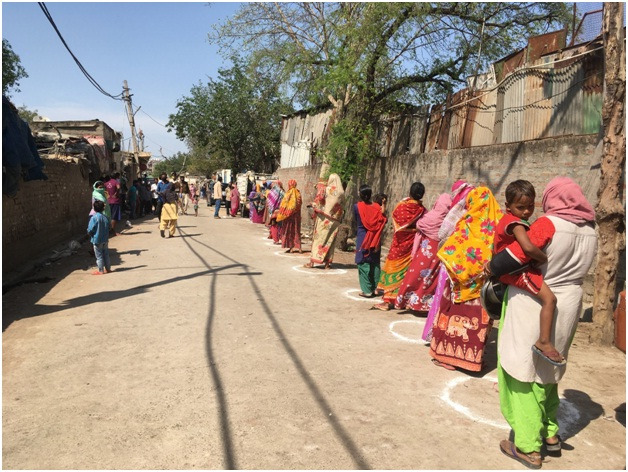
Photo: From the collection of Anumeha Yadav, used with permission.

**Figure 1:**
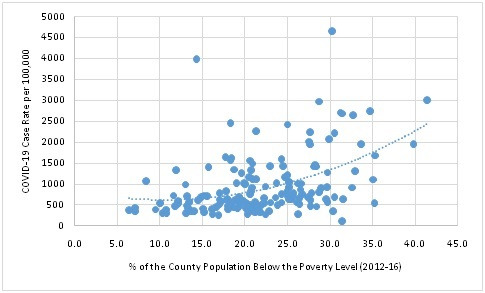
County-level COVID-19 case rates per 100 000 by % of population below poverty level, Georgia, United States.

The Southeastern United States also has been hard hit by COVID-19, with many calling the pandemic more devastating than hurricane Katrina [6]. Georgia, the Southern state where we live and work, is illustrative. Georgia consistently ranks among the top 15 states nationally in numbers of COVID-19 deaths; however, the death burden is highly geographically concentrated within the state. Hardest hit are the poorest, most food-insecure counties having the highest percentages of Black and non-English speaking residents [7]. The most marginalized populations in these counties have poor access to protective equipment, testing facilities, and treatment. These residents cannot afford long periods without jobs or unemployment insurance, and hurried school closures caused acute food insecurity for children. As claims for unemployment insurance mounted during the stay-at-home executive order, the governor precipitously ‘reopened’ the state’s economy [8]. This decision has enabled many of the economically privileged to stay at home and reduce work-related travel [8]. Meanwhile, the poorest residents across the state, who also predominate in “essential industries” [9], have been forced to decide between returning to work with uncertain protections or staying at home without a wage. Case rates now are at an all-time high [7], and at the county level, are highly correlated with five-year estimates for the percentage of the population below the poverty level (**Figure 1**).

Of course, we recognize that lockdowns are necessary to slow viral transmission, and thereby, to manage hospital caseloads. We also recognize that re-opening the economy is critical to mitigate the collateral health and socioeconomic impacts of COVID-19. We also lack the space here to elaborate the nuances of state-level responses to COVID-19 in both countries. However, juxtaposing the overarching responses of India and a Southeastern state in the US reveals how vulnerable the poor are to inadequate planning at both extremes – to abrupt total lockdowns and to precipitous re-openings – in complex, diffuse, and unequal democracies.

Disproportionate effects on the poor under opposing lockdown scenarios raise crucial questions about the balance of powers in diffuse democratic states, the real capacity for coordinated action under crisis, and norms of collective responsibility among citizens. In democracies that uphold equality of opportunity as a constitutional value, abrupt lockdowns and re-openings are unquestionably inhumane. The public mindset that enabled this inhumanity to persist warrants scrutiny. This lackluster public response stems partly from pre-existing blinders to the geographic concentration of inequality in both countries. Lifting these blinders will be essential for civil society to hold states accountable for distributing social protections where and to whom they are most needed, during and beyond COVID-19.
